# Dopa-responsive parkinsonism without cerebellar ataxia in spinocerebellar ataxia 6

**DOI:** 10.1016/j.prdoa.2025.100322

**Published:** 2025-04-02

**Authors:** Shun Yoshida, Toshiyuki Takahashi, Naoki Suzuki, Muneshige Tobita

**Affiliations:** a Department of Neurology, NHO Yonezawa National Hospital, 26100-1, Misawa, Yonezawa, Yamagata 992-1202, Japan; b Department of Neurology, Tohoku University Graduate School of Medicine, Sendai 980-8574, Japan; c Department of Rehabilitation Medicine, Tohoku University Graduate School of Medicine, Sendai 980-8574, Japan

## Abstract

• While SCA6 patients mainly show pure cerebellar ataxia, some cases have parkinsonism.
• In SCA6 patients, levodopa responsivity to parkinsonism is seldom reported.
• We show an SCA6 patient with dopa-responsive parkinsonism without cerebellar ataxia.

## 1 Introduction

Spinocerebellar ataxia 6 (SCA6) is an autosomal dominant spinocerebellar degenerative disorder caused by a CAG repeat expansion. Patients with SCA6 generally manifest pure cerebellar ataxia, however some cases exhibit extra-cerebellar symptoms including parkinsonism [1,2]. In SCA6 patients, sensitivity of parkinsonism to levodopa is seldom reported and some cases showing responsiveness and others not. In a literature survey, only a few cases of parkinsonism without cerebellar symptoms in SCA6 have been reported, moreover all of them showed no improvement with levodopa treatment [3,4].

Here, we describe a SCA6 patient with dopa-responsive parkinsonism without cerebellar ataxia.

## 2 Case report

A 71-year-old man presented with a 2-year history of tremor in his right hand, and 1 year later, he also experienced tremor in his left leg. His mother and maternal uncle exhibited pure cerebellar ataxia onset in their 60 s and were diagnosed with SCA6. He had no history of other diseases or symptoms including constipation, olfactory dysfunction or REM sleep behavior disorder. Neurological examination revealed resting tremor in his right hand and left leg along with right dominant mild cogwheel rigidity in upper limbs without cerebellar ataxia, abnormal eye movements or speech abnormalities (Suppl. Video.). Whole blood cell counts and biochemistry profiles were unremarkable except for elevated HbA1c (7.1 %) and LDL cholesterol levels. Brain MRI showed slight cerebellar atrophy (Fig. A, B, C). ^123^I-labeled N-(3-fluoropropyl)-2β-carbomethoxy-3β-(4-iodophenyl)nortropane (FP-CIT) SPECT imaging revealed symmetrical reduced uptake (Fig. D). Cardiac uptake of ^123^I-Metaiodobenzylguanidine (MIBG) was not reduced (Fig. E). Genetic testing for SCA6 identified a CAG repeat expansion mutation with 22 repeats. Following treatment with carbidopa-levodopa (450 mg/day), his symptoms almost disappeared and the score of MDS-UPDRS Part 3 was improved from 39 to 11 (Suppl. Video.). 2 years after treatment, the dosage of carbidopa-levodopa was reduced to 300 mg/day at his request, then his symptoms deteriorated. When the dose was returned to 450 mg/day, his symptoms were relieved again.

**Fig. 1:**
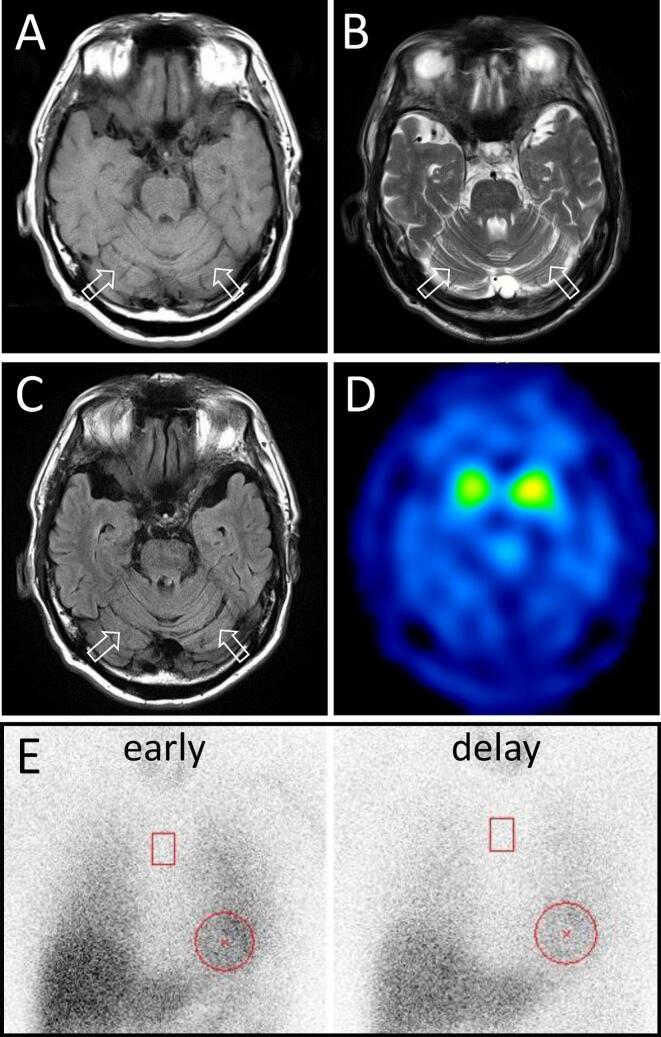
**MRI, MIBG-myocardial scintigraphy and FP-CIT SPECT imaging.** MRI images. A) T1-weighted, B) T2-weighted, C) FLIAR. MRI showed slight cerebellar ataxia (arrow). D) FP-CIT SPECT imaging revealed symmetrical reduced uptake (Specific Binding ratio: right 2.34, left 2.53). E) Cardiac uptake of MIBG was not reduced (heart/mediastinum ratio: early 3.22, delay 3.22).

## 3 Discussion

We report a case of SCA6 patient presenting with dopa-responsive parkinsonism without cerebellar ataxia.

While patients with SCA6 typically manifest pure cerebellar ataxia, some cases exhibit extra-cerebellar symptoms including parkinsonism. Previous reports have indicated that parkinsonism occurs in approximately 20 % of SCA6 patients [1,2]. Neuropathological findings from autopsies of SCA6 patients reveal cerebellar atrophy with severe loss of Purkinje cells, but neurodegeneration of the substantia nigra and striatum has also been described [5,6]. However, several studies using nuclear medicine imaging have shown dopaminergic dysfunction in SCA6 patients regardless of parkinsonism [2]. Furthermore, longitudinal MRI studies have demonstrated a decrease in the volume of the putamen and caudate in SCA6 patients [7].

Although few reports have investigated the levodopa-responsiveness of parkinsonism in SCA6 patients, one case was reported with dopa-responsive parkinsonism, showing a dot-like morphology 18F-dopa PET image similar to Parkinson’s disease or our case [1]. On the other hand, only a few cases of parkinsonism without cerebellar ataxia in SCA6 have been reported, all exhibiting levodopa-nonresponsive bradykinesia-dominant parkinsonism, in contrast to our case [3,4]. This suggests that diffusion of the neurodegeneration in patients with SCA6 could explain variation in levodopa response. Although FP-CIT SPECT of this patient showed symmetrical reduced uptake, his symptoms have a dominant side. However, the dominant side differs between his hands and legs, which may reflect the differences in the lesion sites in the basal ganglia between the right and left. To clarify the distribution of dopaminergic neurodegeneration in SCA6, further accumulation of cases is needed.

Our case did not exhibit cerebellar ataxia until the patient reached their 70 s despite the usual onset of symptoms in SCA6 occur in the late 40 s, and H/M uptake ratio of MIBG-myocardial scintigraphy was not reduced [2]. Although there are no reports on MIBG-myocardial scintigraphy in SCA6, most patients with Parkinson’s disease show a reduced H/M uptake ratio of MIBG-myocardial scintigraphy. While the age at onset of SCA6 within families may vary, the penetrance of SCA6 is nearly 100 % [8]. Considering these facts, it is more likely that parkinsonism in this patient represents a phenotype of SCA6 rather than being idiopathic Parkinson's disease developed before the onset of SCA6. However, we might also take into account the potential influence on the phenotypic expression of the SCA6 pathological CAG expansion in such patients of other coexisting pathogenic variants or polymorphisms in PD related genes considered as risk factors.

This case indicates the variety of clinical symptoms and the possibility of treatment in SCA6. Similar to our case, some SCA6 patients may present with parkinsonism without cerebellar ataxia, and we hope that further accumulation of cases will contribute to elucidating the pathogenesis and identifying biomarkers of levodopa responsivity in parkinsonism associated with SCA6.

## CRediT authorship contribution statement

**Shun Yoshida:** Writing – original draft, Resources, Investigation, Conceptualization. **Toshiyuki Takahashi:** Writing – review & editing, Supervision. **Naoki Suzuki:** Writing – review & editing, Supervision. **Muneshige Tobita:** Writing – review & editing, Supervision.

## Funding

This research did not receive any specific grant from funding agencies in the public, commercial, or not-for-profit sectors.

## Declaration of competing interest

The authors declare that they have no known competing financial interests or personal relationships that could have appeared to influence the work reported in this paper.
